# The CanOE Strategy: Integrating Genomic and Metabolic Contexts across Multiple Prokaryote Genomes to Find Candidate Genes for Orphan Enzymes

**DOI:** 10.1371/journal.pcbi.1002540

**Published:** 2012-05-31

**Authors:** Adam Alexander Thil Smith, Eugeni Belda, Alain Viari, Claudine Medigue, David Vallenet

**Affiliations:** 1 CEA, DSV, IG, Genoscope, Evry, France; 2 CNRS-UMR8030, Evry, France; 3 Université d'Evry Val d'Essonne, Evry, France; 4 INRIA Grenoble-Rhône-Alpes, Team BAMBOO, Montbonnot, France; The Centre for Research and Technology, Hellas, Greece

## Abstract

Of all biochemically characterized metabolic reactions formalized by the IUBMB, over one out of four have yet to be associated with a nucleic or protein sequence, *i.e.* are sequence-orphan enzymatic activities. Few bioinformatics annotation tools are able to propose candidate genes for such activities by exploiting context-dependent rather than sequence-dependent data, and none are readily accessible and propose result integration across multiple genomes. Here, we present CanOE (Candidate genes for Orphan Enzymes), a four-step bioinformatics strategy that proposes ranked candidate genes for sequence-orphan enzymatic activities (or orphan enzymes for short). The first step locates “genomic metabolons”, *i.e.* groups of co-localized genes coding proteins catalyzing reactions linked by shared metabolites, in one genome at a time. These metabolons can be particularly helpful for aiding bioanalysts to visualize relevant metabolic data. In the second step, they are used to generate candidate associations between un-annotated genes and gene-less reactions. The third step integrates these gene-reaction associations over several genomes using gene families, and summarizes the strength of family-reaction associations by several scores. In the final step, these scores are used to rank members of gene families which are proposed for metabolic reactions. These associations are of particular interest when the metabolic reaction is a sequence-orphan enzymatic activity. Our strategy found over 60,000 genomic metabolons in more than 1,000 prokaryote organisms from the MicroScope platform, generating candidate genes for many metabolic reactions, of which more than 70 distinct orphan reactions. A computational validation of the approach is discussed. Finally, we present a case study on the anaerobic allantoin degradation pathway in *Escherichia coli* K-12.

## Author Summary

The discovery of the various metabolic functions catalyzed by enzymes encoded by the genes from the exponentially increasing number of sequenced genomes is one of the main focuses of bioinformatics tools today. However, most of these tools rely on already identified enzyme-coding gene or protein sequence information to predict known enzymatic activities in new genomes. Therefore, they cannot be used to reveal metabolic activities without any corresponding sequenced genes, dubbed “sequence-orphan activities”. In such cases, the best approach is the bioanalysis of target genes by human expert curators, manually integrating so-called “context-based information” (such as gene co-localization on the genome, or the presence of incomplete metabolic pathways) to infer novel functions. Few bioinformatics tools exploit such information and render accessible results in an automated way. Here, we present “CanOE”, a strategy that uses contextual information to propose and rank Candidate genes for Orphan Enzymes in Bacteria and Archaea. Beyond the merit of extending our knowledge and comprehension of prokaryote metabolism, identifying coding genes for sequence-orphan activities opens new opportunities for functional annotation (homology-based transfer made accessible), drug design (new metabolic targets), synthetic biology (new building blocks) and biotechnology applications (new biocatalysts).

## Introduction

Approximately 27% of all enzymatic activities recognized by the IUBMB [www.iubmb.org] are still sequence-orphan metabolic activities (dubbed “orphan enzymes” for short) in the UniProt databank [1], a number that has decreased slowly over the past years [2]–[4]. It would, of course, be too time-consuming and costly to conduct wet-lab experiments to test all known activities against all genes from the exponentially increasing number of sequenced genomes. Instead, bioinformatics tools have been developed in order to help annotate newly sequenced genes and to guide biologists in selecting the right candidate genes for further experimental testing. These tools can be classified into two types: 1) those transferring existing annotations between genes belonging to different organisms on the basis of detected homology (inferred using clues such as high sequence similarity, domain conservation, or feature-based similarities), and 2) those using “context-based” methods capable of inferring functions from existing gene annotations in the same organism, on the basis of detected functional dependence (inferred from clues such as those presented in the following paragraph). Due to the lack of any sequence data, tools based on sequence similarity detection cannot be used to solve the “orphan enzyme” problem, and research has turned to context-based approaches.

Various indicators of prokaryote genes being functionally dependent have been devised in the literature. The foremost of these are collectively termed as “genomic context”, and include gene clustering [5], phylogenetic profiles [6], and gene fusion/fission [7], [8]. “Metabolic context”, for its part, refers in an informal way to the sum of all metabolic knowledge for the genes of a given genomic context. Many ways of exploiting these contextual indicators have been imagined. Manual integration of diverse comparative genomics data sources by expert bioanalysts is an obvious approach, formalized (amongst others) in [5] and [9]. Such strategies have since been put into application in various bioinformatics platforms such as IMG [10], MicroScope [11], 12, the SEED [13] and ERGO [14].

Only a few tools based on these context-based methods have been developed over the past decade with the specific goal of solving the “orphan enzyme” problem. The PathwayHoleFiller-GenomicContext [15] is an improvement over a previous method [16] that allows genomic context similarity measures (gene neighbors, gene clusters, gene fusion, or phylogenetic profile methods, see [17]) as well as metabolic context to be taken into account in a Bayesian classifier. ADOMETA [18] uses various scores (based on gene co-expression, phylogenetic profile similarity, gene clustering, and protein interaction data) integrated using a simple likelihood approach to fill in the missing reactions for three organisms having specifically reconstructed metabolic networks. Yaminishi *et al.* [19] use a kernel approach to integrate two data sources (gene proximity and phylogenetic profiles) to build a global network onto which they project an organism's known reaction set. They then search manually for candidate genes corresponding to orphan reactions based on their operon-like results. Chen *et al.* [20] combine gene sequence similarity and gene proximity across many genomes to establish path-based scores as a functional dependence measure, which is then used to rank candidate genes for pathway holes, including orphan enzymatic activities. Other resources can be exploited manually for finding candidate genes for orphan enzymes using context-based functional dependency measures, such as the STRING [21].

Inspired by the *modus operandi* of human expert research conducted at the Genoscope [22]–[24], we have developed CanOE (Candidates for Orphan Enzymes), an automated strategy that exploits genomic and metabolic contextual information by a graph-based algorithm. This strategy has been integrated into our in-lab genome annotation platform, called MicroScope, and uses its set of expert curated annotations as input, with the objective of improving the reconstructed metabolic networks from the MicroCyc component of the platform [11], [12]. Its results are available via a web interface at the following URL: http://www.genoscope.cns.fr/agc/microscope/metabolism/canoe.php

The principle of our strategy lies in the continuity of previous works [25]–[27]. A first step involves searching for groups of genes corresponding to groups of reactions participating in a same metabolic process. This is done by looking for groups of adjacent genes encoding enzymes catalyzing connected reactions, allowing for gene and reaction gaps. We called the functional units thus identified “genomic metabolons” (in reference to biological metabolons [28]), and they form the basis for the proposition of potential associations (*i.e.* hypothetical annotations) between gene gaps and reactions gaps in the second step. The third step integrates known and potential associations over all available genomes by building gene families and calculating family-reaction association scores. Finally, these scores are used to rank candidate gene-reaction associations. This is particularly interesting when a reaction gap actually corresponds to an orphan enzymatic activity, but can also be used as additional support when transferring annotations on the basis of limited sequence similarity. In this article, we detail the strategy's primary data and operational steps, as well as the evaluation of the performance of our association scores with a benchmarking test. We present a biological case study showing the usefulness of our approach, and finally highlight in which ways our strategy sets itself apart from previous methods.

## Materials and Methods

### Primary genomic and metabolic data

The first step of our strategy requires three types of input data: 1) a gene graph, modeling gene contiguity in a target genome; 2) a reaction graph, modeling the global (*i.e.* pathway- and organism-independent) metabolic network we wish to work with; and 3) the set of all already-known gene-reaction associations, *i.e.* all current functional annotations in the target genome. These data sources are detailed hereafter.

Gene graphs are built separately for each genome from the MicroScope database (1117 available at the time of writing). The gene graph represents all protein-coding genome features (“genes” here) of a single prokaryote organism as vertices. In this work, we use gene contiguity as an indicator of functional dependence. Immediately consecutive genes are thus connected by edges, ignoring gene transcription direction and intergenic distance, as bidirectionally-translated operons have been observed [29], [30]. We thus are independent of operon, regulon, stimulon and über-operon definitions [31], though our metabolons will be still able to capture some parts of such structures.

The reaction graph represents metabolic reactions as vertices. We link two reactions by an edge when the product of one is a substrate of the other. However, to avoid the high connectivity problems that are common when building such metabolic networks, we limited such shared compounds to “main compounds”, *i.e.* metabolites deemed biologically relevant to both reactions in at least one metabolic pathway (for example, phosphoenolpyruvate, but not water, in the glycolysis pathway). Main compounds are arbitrarily defined as such by biochemists on the basis of atom-tracing experiments, molecular structure conservation, or other data. The modeled reactions were extracted from MetaCyc 15.0 [32], but any other generalist metabolic database (preferentially one containing main compound data) can be used (*e.g.* Rhea [33], KEGG reactions [34]…). The metabolic network is global, as it contains all known metabolic reactions and is not split into separate, disconnected pathways. It is thus not organism-specific, guaranteeing maximal metabolic freedom.

Finally, we retrieved functional annotations from the MicroScope platform (and in the case of *Escherichia coli* K-12, additionally from EcoCyc [35]) to benefit from its high level of expert manual curation. The MicroCyc component of MicroScope gathers a set of Pathway Genome DataBases (PGDBs) which were built using the Pathway Tools software [36] and with the MetaCyc database [32] as a reference. Gene-reaction associations are extracted from these PGDBs and used to link elements from the gene graph to those of the reaction graph. This creates a new graph, called the “data graph”, which has two types of vertex (genes and reactions) and three types of edge (gene-gene edges, reaction-reaction edges, and gene-reaction edges). The previously described gene-reaction associations are flagged as “**Known**”, as they correspond to the current state of biological knowledge. The metabolic network is thus populated specifically for each organism by reactions known to be catalyzed within them. It should be noted that multiple reactions can be linked to a same gene (*e.g.* bi-functional genes or enzymes with wide substrate specificity), and conversely, multiple genes can be linked to a same reaction (*e.g.* enzymes with several subunits). Details on graph construction can be found in [Text S1].

### Finding candidate genes for orphan enzymes

Two kinds of “reaction knowledge hole” can be formalized in metabolic networks [37]. The first kind is the **gap reaction**, *i.e.* a missing reaction in an organism-specific metabolic network reconstruction whose presence appears necessary for the network to be complete (without spurious dead-end metabolites). Basically, no experimental results necessarily confirm its presence in an organism, but metabolic context within the organism suggests it. The other kind of “reaction knowledge hole” is the **orphan reaction**, *i.e.* an enzymatic activity thought to be present in an organism (preferably with experimental evidence) but without any known coding genes. Reactions can be orphans in a specific target organism (**local orphan**), or for all known organisms (**global orphan**). In this article, we work exclusively with prokaryote organisms from the MicroScope platform; a reaction is thus considered as a global orphan when it has no known coding genes in any of the platform's prokaryote organisms (even though it may have coding genes in eukaryote species). In an organism-specific metabolic network, global orphan reactions (if present) may appear as gap reactions. On the other hand, gap reactions may be either local or global orphan reactions.

The CanOE strategy will first detect potential gap reactions by computing metabolons in a global metabolic network populated by reactions known to be catalyzed in the target organism. It will then propose candidate genes for these gap reactions, be they local orphan reactions or global orphans across all of MicroScope's genomes.

#### Building metabolons

The previously built data graph is processed by a modified version of the Common Connected Component Partitioner (C3P) [26], [27]. This exact algorithm partitions the data graph into a set of largest possible subgraphs (Common Connected Components, CCCs) which verify that a) their gene graph part is a connected component (*i.e.* any vertex can be reached from any other vertex by a path in the graph), b) their reaction graph part is a connected component, c) each gene is connected to at least one reaction and vice-versa, and d) that the final subgraph is built on a maximal number of gene-reaction associations. Let us note that by construction, a given gene-reaction association can be present in only a single metabolon, though a gene or reaction may be present in multiple metabolons due to it belonging to multiple gene-reaction associations. Metabolon gene and reaction gaps are technically introduced by using the g-partial transitive closures of the gene and reaction graphs, where g is the number of gaps (*i.e.* the number of intermediate vertices) allowed. Metabolon gaps are recovered by a CCC post-processing step, detailed in [Text S2]. Metabolon reaction gaps determined in this way may correspond to “gap reactions” as described above, or they can correspond to reactions catalyzed by genes located elsewhere on the genome. In the CanOE strategy, the g parameter was empirically fixed to 3 for the metabolon gene gaps and to 2 for metabolon reaction gaps. Indeed, the average size of *E. coli* multi-gene transcription units defined in RegulonDB [38] is 3.2 genes, and 90.5% of all known transcription units could be covered by using a gene gap parameter of 3 even in the worst case scenario (*i.e.* two genes separated by 3 gene gaps); furthermore, the mean distance between any two vertices in our global metabolic network is 1.9 intermediate vertices, and 71.3% of all reaction vertex pairs can be joined using a reaction gap parameter of 2. Finally, we set the minimal number of genes, reactions and Known gene-reaction associations for a CCC to be retained to 2, 2 and 2, respectively.

**Figure 1:**
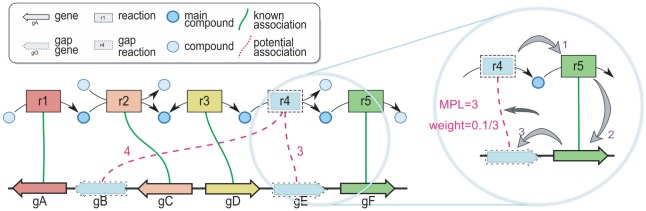
An example metabolon. One genomic metabolon containing a) 6 genes represented by arrows (gA–gF) of which two are gaps (gB and gE); and b) 5 reactions represented by rectangles (r1–r5) of which one gap (r4). Sequential genes are joined. Reactions are joined together by shared main compounds, *i.e.* compounds relevant to at least one biological pathway, represented here as deep blue circles. Gene annotations are materialized by Known gene-reaction association edges, in green. Potential gene-reaction associations are proposed between gene and reaction gaps, in dotted pink. For each Potential association, a Minimal Path Length (“MPL”) is computed. The MPL is defined here as the minimal number of non-Potential edges that must be walked to join a gene gap to a reaction gap (illustrated in the detail shot). Each Potential association is then weighted by α_potential_/MPL, where α_potential_ is arbitrarily set to 0.1.

Each CCC (with recovered gaps) corresponds to one genomic metabolon, and is saved in the MicroScope database. “**Potential**” gene-reaction associations are then generated between metabolon gene and reaction gaps and are held for further analysis in the next step. In the example metabolon illustrated in [Figure 1], reaction r4 and gene gB are gaps, and a Potential association is proposed (materialized by a dotted pink line). The strength of our method lies in the fact that these associations are generated without use of sequence data, allowing it to propose candidate genes even for sequence-orphan reactions.

#### Weighting and scoring proposed gene-reaction associations

For each Known or Potential gene-reaction association in a metabolon, we compute a score based on their Minimal Path Length (hereafter MPL), which is defined as the length of the shortest path traversing the metabolon graph between the concerned gene and the concerned reaction (details given in [Text S3]). To obtain a meaningful context-based measure of distance between the two, only Known edges may be traversed between genes and reactions (all gene-gene and reaction-reaction edges are allowed); furthermore, if the association for which the MPL is being calculated is already Known, its own edge is not traversed, ensuring that the MPL captures a measure of local gene/reaction colinearity. Establishing the MPL is illustrated in the focus inset of [Figure 1], between gene gE and reaction r4. The weight w_G,R_ of a (gene G, reaction R) edge is set to α/MPL, where α is a constant factor specific to the type of association, intuitively and arbitrarily set to favor Known associations over Potential ones (typically: α_known_ = 1 = 10*α_potential_). In metabolons with multiple gene and/or reaction gaps, these weights allow us to prioritize candidate genes that are topologically close to the metabolon reaction gap. This biological prior was established by observing the distribution of the MPL measure for all Known associations, given in [Figure S1].

Gene-reaction associations are then evaluated using “gene-level association scores”, *i.e.* scores measuring the strength of the association of genes to reactions or reactions to genes, given all the metabolon evidence across a given genome. For a given gene G in organism O, for a given reaction R, the gene-to-reaction score is the ratio of the weight of the association (G,R) over all the associations (G,*) for all reactions across the entire metabolic network:
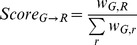
Likewise, the reaction-to-gene score is the ratio of the weight of the association (G,R) over all the associations (*,R) for all genes in the target Organism O:
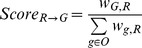
These descriptors evaluate the association strength between a given gene and a given reaction, taking into account other associations that both belong to. They can thus be used to rank candidate reactions for given genes, or candidate genes for given reactions. We will compare them to equivalent family-level descriptors in order to show that integrating results over all organisms is worthwhile.

#### Integrating over all organisms

**Figure 2:**
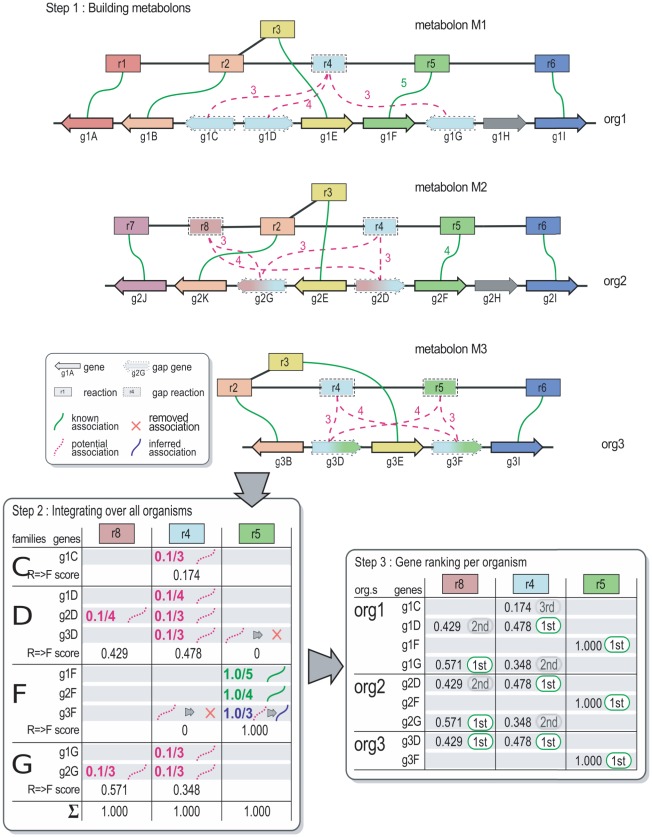
The CanOE strategy. Step 1: CanOE locates three metabolons (M1, M2 and M3) located in 3 different prokaryote genomes (org1, org2, org3). Relevant minimal path lengths (MinPathLengths, MPL) are given for their associations. For example, gene g3D has a Potential association with reaction r4, with a corresponding MPL of 3. Non-metabolic genes are gray and are not candidates for Potential associations. Gene families are materialized by gene names (*e.g.* genes g1D, g2D and g3D all belong to family D). Step 2: The table summarizes how metabolons are integrated over all organisms. Separate gene-reaction associations are counted for each gene family- reaction pair, and are weighted by the ratio of a constant association type-specific α factor over the inverse of their MPLs. If a family contains Known (solid green curve) and Potential (dashed pink curve) gene-reaction associations with a same reaction, the Potential associations are transformed into “Inferred” associations (solid blue curve), and other now impossible associations are removed (red crosses). Association scores can then be calculated for each family-reaction pair. Here, reaction-to-family scores are given as the fraction of weighted associations that a given family has for a given reaction, over all the weighted associations the given reaction has. For example, family G (to which gene g1G belongs to) has a R = >F score of (2*0.1/3+0.1/4)/(5*0.1/3+0.1/4+0) = 0.348 for reaction r4. Step 3: Finally, gene-reaction associations are ranked for each reaction in each organism according to their corresponding family-level score. Gene g1G is thus the second-best candidate for reaction r4 in organism 1, but is the best candidate for reaction r8.

Even if the existence of a metabolon is good support for a Potential (gene gap, reaction gap) association, it is difficult to evaluate how credible the proposition is without checking for its presence in several other organisms. The next step of our strategy is therefore dedicated to the integration of metabolon data across all the prokaryote genomes stored in MicroScope, with the idea that metabolon structures should be partially or exactly conserved across several organisms. [Step 1 of Figure 2] is an illustrative example of this approach, representing three metabolons with common reactions from three different organisms. The MPL measure is given for Potential associations and Known associations of interest (*e.g.* 3 for g1G and r4 in metabolon M1).

To integrate over all organisms, we built vertical relationships between genes of different organisms with a home-tailored gene clustering procedure based on the OrthoMCL algorithm [39] which uses protein sequence similarity as a metric between genes, as described in [Text S4]. These families are materialized in the example from [Step 1 of Figure 2] by the gene labels, *e.g.* gene g2D belongs to family D.

We analyzed the combined Gene Ontology annotations [40] of any Interpro domains [41] the families contained. Whenever possible, gene families that could be established as non-metabolic (*i.e.* not encoding enzymes participating in metabolism) according to the GO terms were flagged as such (for details see [Text S5]). All Potential gene-reaction associations from these families were then removed from further analysis, as we are only concerned with metabolic reactions. This was particularly useful in eliminating candidate associations from large conserved protein families like ABC transporters and various regulators which are commonly included within metabolic operons.

In some cases, CanOE may find in one metabolon a Potential association between a given reaction and a gene belonging to a family, within which another member is already known to be associated to this reaction. For example, in metabolon M3 of [Figure 2], gene g3F has a proposed Potential association with reaction r5, whereas g2F, another gene from family F, is already associated to r5 by a Known association in metabolon M2. We consider this sufficient evidence to transfer the annotation from g2F to g3F, labeling the (g3F, r5) association as “**Inferred**” rather than Potential (materialized by a solid blue arc in Step 2). Inferred associations are weighted like Known associations (α_inferred_ = α_known_). At the same time, g3F and r5 cease to be gaps in metabolon M3, thus removing any other Potential associations concerning them (these are (g3F, r4) and (g3D, r5), materialized by red crosses in step 2). This is useful in eliminating some false positive Potential associations generated by CanOE. We argue that this annotation transfer is safer than traditional transfer on the basis of sequence similarity, as we have here the additional condition that the metabolic context must be sufficiently conserved to be able to propose the corresponding Potential association.

In order to quantify the support for a family-reaction association, we calculated three family-level descriptors reminiscent of the previously-defined gene-level association scores, that take into account all Known, Inferred and Potential gene-reaction associations across all organisms. For a given association between family F and reaction R, the Coverage (*Coverage _F,R_*) is the fraction of the genes from the family associated with the given reaction, whatever the association category:
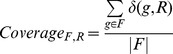
Where δ(g,t) is 1 when gene g is associated to reaction t (by Known, Potential or Inferred associations), and is 0 otherwise; |F| is the number of genes in family F. The Family-to-Reaction association score (*Score _F→R_*) is the fraction of the weight of all gene-reaction associations possessed by the family F that involve the given reaction R:
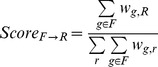
The Reaction-to-Family association score (Score *_R→F_*) is the fraction of the weight of all gene-reaction associations possessed by the reaction R that involve the given family F:
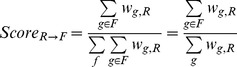
Examples of reaction-to-family association scores are given [Step 2 of Figure 2].

Each of these scores reflects the strength of the association between a given reaction and a given family: the Coverage gives a general idea of the association by counting the number of members of the family that are associated the reaction; the two selectivity scores are finer and capture the asymmetry of family-reaction associations. It is important to note that these family-level descriptors differ from the gene-level descriptors in three ways: 1) they use Known, Potential and Inferred gene-reaction associations, whereas gene-level descriptors only use Known and Potential associations; 2) less Potential associations are available to family-level descriptors as the inference step has removed some of them; and most importantly 3) family-level descriptor values are not calculated per genome but for all MicroScope-available genomes.

#### Candidate gene ranking

Family-level association scores integrate information across several organisms about the association strength between member genes and target reactions. Our objective is to make both gene- and family-level scores available to bioanalysts annotating genes as indicators of how likely their target genes encode enzymes catalyzing the target reactions. It is then possible to rank candidate reactions for a given gene, or candidate genes for a given reaction, in each organism using either gene-level or family-level scores. [Step 3 of Figure 2] shows the ranking of candidate genes according to the reaction-to-family association score, for each (organism, reaction) couple. In order to evaluate how informative the generated ranks were, we carried out the following benchmarking experiment.

### Benchmarking

In our benchmarking experiment, we considered the set of all metabolic reactions having at least one Known gene-reaction association involved in a metabolon (since the method does not make predictions for genes and reactions not involved in metabolons). For each reaction from this set, we removed all the gene-reaction associations involving that reaction in all organisms (effectively rendering it a sequence-orphan reaction), and recalculated all gene- and family-level association scores, for which we consider the rank of the genuine gene-reaction associations. Results were pooled across all reactions from the set.

A recovered gene-reaction association is considered as a positive hit when its rank (according to a chosen score) is below a certain threshold k. All recovered associations can be declared as positive hits by taking k = ∞.

For a level of k, we defined true positive associations (TP) as the number of genuine gene-reaction associations that were recovered in the experiment in respect to the original CanOE run, false negative associations (FN) as those that were not recovered, and false positives (FP) as Potential associations that were proposed that did not correspond to Known associations in the original run. We then classically compute the **recall** (or sensitivity) as the fraction of recovered associations (TP/(TP+FN)) and the **precision** as the fraction of correctly predicted associations (TP/(TP+FP)).

In order to gauge how indicative our family-reaction descriptors are of gene-reaction association strength, we examined the evolution of the recall and the precision while varying the rank threshold k (*i.e.* keeping only the k-best associations for each gene), thus generating a precision-recall curve for each score.

## Results

### General results

The genomes of 1,090 prokaryotes from the MicroScope platform produced a total of 61,670 metabolons, leading to an approximate average of 57 metabolons per organism (see [Figure S2]). *E. coli* K-12, at 105 metabolons, is comparatively rich. A brief analysis showed that 78 of these metabolons (74%) shared at least two genes with operons defined by RegulonDB [38]. All in all, the density of genomic metabolons is consistent with previous findings, given the current state of functional annotation amongst bacterial genomes [25].

These organisms contained a total of 4,646,851 genes, of which 1,088,330 (32.6%) had metabolic annotations (*i.e.* genes coding for enzymes represented in the MicroCyc database). The metabolons themselves covered 215,968 of these genes (19.8%). When considering the well-annotated genome of *E. coli* K-12, 1,441 out of 4,414 genes (30.7%) were annotated with metabolic activities, and 399 of these (27.7%) were in a metabolon. The per-organism gene coverage of the metabolons varies between 2.5% and 7.5% as shown in [Figure S3]. The distributions per phylum are given in [Figure S4]. The genes were grouped into 8,629 gene families by our clustering method, of which 616 (7.1%) were declared non metabolic.

Our local installation of the MetaCyc database (version 15.0) contains 9,531 reaction instances. Using pathway-specific main compound definitions, reaction-reaction edges were added between these. A total of 5,157 reactions were connected in this way in our global metabolic network. Of these, an impressive 2,839 (55.1%) are sequence-orphan activities across all MicroScope prokaryote genomes. 1,626 (31.5%) reactions were found in at least one metabolon, showing that the coding genes of almost two thirds of available reactions are never clustered throughout prokaryote genomes, and thus cannot be captured by metabolons based on simple gene neighborhood. Furthermore, only 104 (6.4%) of these were global orphans, showing that well-known metabolic reactions are generally surrounded by other well-known reactions. Finally, at the time of writing, 72 of these had potential gene candidates, and 50 of these had candidate genes belonging to a gene family, allowing the calculation of the family-level association scores. Only one prokaryote orphan reaction was found with candidate genes in *E. coli* K-12, and is described in the case study section. The list of all proposed candidate genes for all found global orphan enzymatic activities is available from the CanOE main web page by simply selecting the “Consult global orphan reactions” radio button and clicking “Go!”. A manual bioanalysis of these cases is summarized in [Text S6]. We determined that 31 of the global orphan reactions may have plausible candidate gene predictions. Of particular interest, 20 of these have good CanOE-independent supporting evidence (*e.g.* circumstantial literature, sequence similarities with enzymes of related reactions, predicted domains…).

### Benchmarking results

To evaluate the CanOE strategy in a systematic way, we undertook a benchmarking experiment allowing us to a) quantify how well Known gene-reaction associations were recovered after having been removed from the input data, and b) show how informative our gene- and family-level scores are. 1,469 of the MetaCyc reactions that had been observed in metabolons were sequentially rendered orphan in our benchmarking experiment.

The global (*i.e.* for a rank cut-off of k = ∞) gene-level recall is 80.5%, meaning that over four out of five Known gene-reaction associations that were removed by the orphan experiments were successfully recovered. The global gene-level precision is 45.2%, meaning that a little less than half of all Potential associations generated in the experiments are indeed true associations. At the family level, the global recall is 80.6% and the precision is 45.4%, showing a very slight global improvement imputable to family-wise association redefinition.

**Figure 3:**
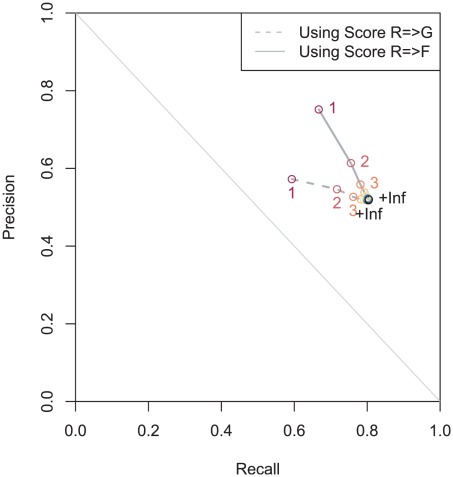
Precision-recall curves for reaction-to-gene and reaction-to-family association score ranks. Evolution of recall and precision when applying an increasing rank cutoff (values indicated by colors) to the reaction-to-gene (dashed lines) and reaction-to-family (solid lines) association scores. Values hardly vary after the 10 first ranks, indicating that almost all true hits have at worst rank 10, though good performances are observed amongst the first 4 ranks.

However, more importantly, we show in the precision-recall curves of [Figure 3] that the family-level Score*_R→F_* rank outperforms the gene-level Score*_R→G_* rank. We can observe that the precision can be increased with minimal impact on the recall by keeping no more than the best 3 or 4 candidates according to the former, when the recall is more heavily impacted for less precision improvement in the latter. Individual TP, FP, FN counts can be found in [Table 1, Table 2]. This illustrates a definite advantage of exploiting integration over all available organisms to help rank proposed gene-reaction associations, even if the maximal precision and recall values (obtained when considering all propositions) hardly differ. The precision-recall curve and its associated tables for Score*_F→R_ and* Score*_G→R_* can be found in [Figure S5, Table S1, Table S2], showing that it is also informative, though with a lower performance. To be comparable to self-rank tests as in [42], we show the fraction of recovered TP associations as a function of a maximal rank cutoff in [Figure S6]. Over 99% of TPs are found amongst the first 5 ranks, outperforming ADOMETA [18], consistent with results obtained by Chen et al. [20], though possible ranks go up to around 80.

**Table 1:** Precision and recalls for the benchmarking of the reaction-to-gene association score.

k	True Positives	False Positives	False Negatives	Recall	Precision
1	104 347	77 941	71 563	59,32%	57,24%
2	126 146	104 762	49 764	71,71%	54,63%
3	133 976	120 372	41 934	76,16%	52,67%
∞	141 394	131 562	34 516	80,38%	51,80%

**Table 2:** Precision and recalls for the benchmarking of the reaction-to-family association score.

k	True Positives	False Positives	False Negatives	Recall	Precision
1	118 172	39 019	58 990	66,70%	75,18%
2	133 782	84 178	43 380	75,51%	61,38%
3	138 426	109 390	38 736	78,14%	55,86%
∞	142 545	131 562	34 617	80,46%	52,00%

We conclude that these descriptors are sufficiently informative for use by biologists, biochemists and bioanalysts in determining which candidates are the best bets to test experimentally when considering potential annotations with orphan reactions.

### Case study

**Figure 4:**
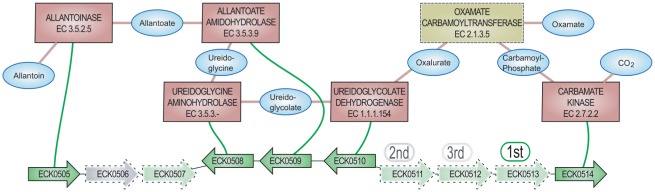
*E. coli* K-12 anaerobic allantoin degradation metabolon. Case study metabolon for the anaerobic degradation of allantoin in *E. coli* K-12. Genes are shown as arrows with their MicroScope labels, reactions are shown as rectangles with their MicroCyc names and EC numbers. The orphan activity, oxamate carbamoyltransferase, is shown as a yellow box. Main compounds of interest are shown for reference and readability. Oxamate carbamoyltransferase is the only reaction gap in this metabolon. Ranks based on reaction-to-family association scores are shown for the possible three candidate genes for it (ECK0506 is a non-metabolic gene, and ECK0507 is already annotated with a Known association in another metabolon, hence they cannot be candidates for this activity).

To illustrate the usefulness of our method, we investigated a predicted metabolon in *E. coli* K-12 containing a prokaryote orphan reaction with candidate genes [Figure 4]. This metabolon is composed of 6 reactions covering the complete pathway for the anaerobic degradation of allantoin, in which two reactions are orphans in *E. coli* according to the EcoCyc resource [35]: oxamate carbamoyltransferase (OXTCase, global prokaryote orphan) and carbamate kinase (CKase, local orphan). In the CanOE metabolon [Figure 4], the CKase is shown to be catalyzed by the ECK0514/*ybcF* gene: this association is absent from EcoCyc, despite the latter being a heavily-curated resource, but is supported by the MicroScope annotation of this gene which shares more than 50% amino acid identity with an experimentally-validated CKase from *Pseudomonas aeruginosa* (P13982 UniProt entry). This first point demonstrates how the CanOE strategy can aid bioanalysts to confirm putative annotations for local orphan reactions by automatically mining the wealth of a metabolic context.

The second missing activity in *E. coli* K-12 (the OXTCase) has yet to be associated to any genes in any organism and is thus a global orphan activity, despite its presence having been biochemically demonstrated in *Streptococcus allantoicus* [43], and even reported in *E. coli* [44], [45]. The CanOE metabolon bearing this reaction [Figure 4] contained 5 gap genes (ECK0506-507 and ECK0511 to 0513) that could serve as candidate genes. The first one, ECK0506/*ybbY*, belongs to an Intepro family defined by the presence of a permease domain and is annotated as a putative purine permease according to the UniProt resource [1]. This gene was declared non-metabolic by CanOE and thence not considered as a potential candidate for the OXTCase activity. However, the purine permease function is quite consistent with trans-membrane transport of allantoin or another intermediate of purine metabolism, of which allantoin degradation is a part.

The second gene, ECK0507/*glxK*, was experimentally demonstrated to encode a glycerate kinase involved in the aerobic degradation of allantoin via glyoxylate metabolism [45]. This gene was a non-gap member of a neighboring CanOE metabolon (genes ECK0500 to ECK0507) that contains three known gene-reaction associations involved in glyoxylate degradation. ECK0507 was thus not be considered by our strategy as a candidate for OXTCase activity either. It may be interesting to note that the genes behind both the anaerobic and aerobic degradation of allantoin are neighbors in E. coli K12's genome.

The remaining three candidate genes (ECK0511 to ECK0513) were ranked at the family-level using CanOE-generated family-level scores; values are given in [Table 3]. ECK0513/*ylbF* and ECK0512/*ylbE* belong to two distinct Pfam families harboring domains of unknown function (DUF2877 and DUF1116, respectively) which are conserved over half a thousand proteins from other organisms; either could be good candidates. We have noticed that the gene sequence of ECK0512/*ylbE* presents a frameshift which is absent in all other sequenced *E. coli* strains and may be due, according to UniProt, to a sequencing error. The sequence analysis of the third candidate gene (ECK0511/*fdrA*) gave more clues about its potential molecular function. Indeed, this gene belongs to a family defined by the presence of a conserved domain (PF00549 Pfam domain), many members of which are annotated as CoA-ligase enzymes. The reaction mechanism of the OXTCase activity resembles in no way that of a CoA-ligase activity, suggesting that this gene does not catalyze the former activity. We hypothesize that, if the Pfam assignation is correct, this gene encodes a CoA-ligase which transfers a coenzyme A group to the oxamate produced by the OXTCase enzyme for its degradation by a yet-unknown catabolic pathway (oxamate is currently a dead-end metabolite in the *E. coli* metabolic network).

**Table 3:** Candidate genes for oxamate carbamoyltransferase activity in *E. coli* K-12.

Gene label	Family ID/Size	Coverage _F,R_	Score _F→R_/rank	Score _R→F_/rank
ECK0511	647/133	100,00%	0.9699/2nd	0.334/2nd
ECK0512	639/130	100,00%	0.9701/3rd	0.252/3rd
ECK0513	618/129	100,00%	0.9705/1st	**0.344/1st**

None of the three candidate genes (ECK0511 to 0513) share any significant sequence similarities with known carbamoyltransferases. Thus, the candidate genes proposed by CanOE suggest that the OXTCase activity may be catalyzed by a previously-unknown family of carbamoyltransferases. This hypothesis is consistent with a recent study which did not observe any OXTCase activity for gene ECK2866/*ygeW*, the last *E. coli* K-12 member of the known carbamoyltransferase family whose function remains to be elucidated [46]. Starting with the best-ranked CanOE candidate (ECK0513/*ylbF*), protein expressions and biochemical assays are currently under way.

Needless to say, given that the genomic metabolon-defining step of the CanOE strategy is based on the *modus operandi* of bioanalysts, any respectable bioanalyst could propose candidates genes for reaction gaps after a manual examination of our metabolons. However, the added value of CanOE results are multiple: 1) metabolons are established by an automated procedure, and are distinguished as functional units of a target genome, saving the bioanalyst the effort of locating and building it in his mind; 2) Potential gene-reaction associations are also generated automatically, akin to the hypotheses a bioanalyst formulates during his work; 3) results are integrated across a thousand genomes, a very difficult task for a human to undertake, in the form of a few scores and ranks that can be easily interpreted; 4) all CanOE results are available to the bioanalyst community via a MicroScope platform web interface, making them easily exploitable.

## Discussion

Due to its independence to sequence similarity in its first step and its usage of genomic and metabolic contexts, our CanOE strategy is capable of detecting reaction gaps and proposing candidate genes for them, even in the case of orphan reactions. Calculated metabolons have a relatively good genome coverage (approximately 5% of genes, out of an estimated possible maximum of 30%) and even better metabolic network coverage (1,628 out of 5,157, *i.e.* 55%). Results are integrated over more than 1,000 prokaryote organisms. We show in a benchmarking experiment that our family-based association scores are informative for the selection of the most promising gene candidates for orphan enzymatic activities; indeed, when keeping the 3 best-ranked associations, precision is 52% for a recall of 76.5%. Out of 72 global orphans with CanOE-proposed candidate genes, 20 of these seemed particularly promising after manual bioanalysis. Even the highly-curated *E. coli* K-12 genome yielded one orphan reaction with candidate genes, for which biochemical testing is under way.

Other methods exploiting genomic and sometimes metabolic context have been designed in previous works to propose candidate genes for orphan enzymatic activities. Most of them [15], [19], [20] are pathway-dependent in that they require the presence of a predicted pathway (*i.e.* in which at least one reaction is assigned to one gene in the target organism) to propose candidate genes for the remaining unassigned reactions of that pathway. ADOMETA [18], on the other hand, is not a pathway-dependent method, but orphan reactions must be explicitly described in the organism-specific metabolic reconstructions to be used as targets for candidate genes. Furthermore, ADOMETA requires a filtering step to reduce their metabolic network connectivity: they remove reaction-reaction edges corresponding to the 15 most connected compounds, taking the risk of losing important edges. In comparison to these methods, CanOE uses main compounds defined in metabolic pathways to create a sparser and more biologically relevant global network. This network is thus pathway-dependent in its scope (no reactions not assigned to a pathway are included in it), but is independent in its use (*i.e.*, metabolons can cross multiple pathways). This scope currently limits us to 2,839 of all MetaCyc prokaryote orphan reactions, though this should improve as additional metabolic data is integrated into the MetaCyc database. Also of note is the fact that our strategy predicts gap reactions under the constraint of necessarily anchoring a metabolic context by at least two known reactions to two co-localized genes, making the approach more robust in respect to the quality of the organism's predicted metabolic network.

Unlike previous methods, CanOE is an approach that explicitly integrates its results across several organisms in order to refine and rank its predictions. In the approach developed by Yamanishi *et al.* [19], candidates are not prioritized, and results across a small number of organisms must be integrated manually. Methods like ADOMETA and the STRING [18], [21] do propose functional association scores that can be used to rank candidates on a per-organism basis; the PathwayHoleFiller-GC method [15] gives an association probability extracted from a Bayesian network. However, even though phylogenetic profile similarity measures are used as input, the results of these methods do not explicitly take into account results across many genomes. Making our family-level selectivity scores available at gene level allows CanOE to have greater power distinguishing false positive associations by favoring conserved associations, as we have shown in our benchmarking experiment.

In the CanOE strategy presented here, we exploit the simplest of genomic context indicators, gene neighborhood, the use of which relies on the observation that genes involved in a same biochemical process tend to cluster on prokaryote genomes, forming operons or regulons. We chose this indicator as it is the most visible and easiest to interpret, and has been shown to outperform other genomic context indicators [47]. It does, however, have some shortcomings. In our graph-based algorithm, gene gaps are located, by construction, between non-gap genes within the metabolon; therefore, genes flanking the metabolon are not proposed, excluding possible interesting candidates. However, systematically proposing all metabolon-flanking genes as candidates leads to many false positive propositions. So far, we argue that our necessity of anchoring a metabolon between at least two genes is a guarantee of the quality of the metabolon; in the worst cases, the manual bioanalysis of a metabolon *in situ* on the genome may reveal interesting candidates nearby.

Another limitation of our approach is that genes participating in a same biological process might not be clustered on the chromosome because they are linked by other, more complex regulatory mechanisms. In this case, CanOE will obviously not be able to find a metabolon, and hence be unable to propose candidate associations. The previous works discussed above have the advantage of being able to propose candidate genes that are not clustered on the chromosome thanks to their use of other functional dependence measures (such as gene co-regulation, phylogenetic profile similarity, co-citation…). We plan to include additional genomic context indicators within our strategy to extend its scope, if they prove to be informative. For example, it would be possible to use phylogenetic profiles calculated across all organisms, linking genes with high similarities. This modification would allow metabolons to span groups of genes scattered throughout the genome, capturing larger biological processes and opening up new possibilities for gene candidates.

Our metabolons currently cover over 1,060 prokaryote organisms, which is much more than previous strategies achieved [18], [19], [25], and results are integrated *a posteriori* using gene families. The actual use of gene families for functional annotation remains debatable [48]. Here, we argue that our gene families are not designed to serve as accurate representations of true ortholog families, but only as a means of reinforcing CanOE-proposed associations across several genomes, associations which were, after all, generated based on data other than sequence similarity. Another possibility would be to directly integrate gene-reaction association scores and gene-gene sequence similarity scores rather than use gene families, if computational tractability problems can be overcome.

CanOE results are of interest to the bioanalyst community at four different levels. Firstly, the many metabolons generated by CanOE are independent functional units of a target genome, and each can be represented as easily interpreted graphs. As such, they can be used as an aid to annotation. Secondly, a large fraction of these metabolons are exploited to automatically generate potential gene-reaction association hypotheses, with informative cross-organism integrated scores and ranks to further guide manual annotation. Thirdly, some of these are automatically transformed into Inferred associations, thus helping with automated functional transfer. Finally, a small number of the generated associations concern reactions that are sequence-orphan activities, and are thus of paramount interest; being automatically created, bioanalysts can focus on these specific cases. The web interface allows MicroScope platform users to exploit CanOE results to each of these aims. Altogether, these four levels should help metabolons become a reference in annotating prokaryote genomes. Indeed, it is our hope that this strategy will be employed in wider, systematic enzymatic activity screenings; interacting with projects such as COMBREX [49] and the Enzyme Function Initiative [50] should be productive. Iteratively computing gene-reaction association predictions before validating or invalidating them in wet-lab assays should gradually help cover the metabolism of any prokaryote genome.

## Supporting Information

Figure S1 **MinPathLength distribution.** The number of Known associations for each distinct MinPathLength value (with an imposed maximum of 10) are shown. Note that MPL values of 2 are only possible for Known associations involving multifunctional genes or reactions catalyzed by several gene products (*i.e.* enzymes with several subunits).
(TIFF)

Figure S2 **Per-organism metabolon count distribution.** Distribution of metabolon counts per organism. Global average is approximately 56 metabolons per organism. *E.coli* K-12 is very metabolon-heavy with 105 metabolons.
(EPS)

Figure S3 **Per-organism gene coverage distribution.** Distribution of the per-organism fraction of metabolon genes over all genes. Global average is 4.8%. In contrast, 9% of *E. coli* K-12 genes are in a metabolon.
(EPS)

Figure S4 **Distribution of prokaryote organisms from the MicroScope database per phylum.** The number of MicroScope organisms for each phylum is given. The green fraction of each bar represents the number of organisms that were found to contain metabolons.
(TIFF)

Figure S5 **Precision-recall curves for gene-to-reaction and family-to-reaction association score ranks.** Evolution of recall and precision when applying an increasing rank cutoff (values indicated by colors) to the gene-to-reaction (dashed lines) and family-to-reaction (solid lines) association scores. Values hardly vary after the 10 first ranks.
(EPS)

Figure S6 **“Self-rank” cumulative True Positives.** Evolution of ratio of true positives over maximal number of true positives when applying an increasing rank cutoff to the reaction-to-family association score. Increase in this fraction is hardly visible after the 20 first ranks, indicating that almost all true positives have at worst rank 20 according to this score, though a vast majority (97.1%) have at worst rank 3.
(EPS)

Table S1 **Precision and recalls for the benchmarking of the gene-to-reaction association score.**
(XLS)

Table S2 **Precision and recalls for the benchmarking of the family-to-reaction association score.**
(XLS)

Text S1 **Gene and metabolic graph building.**
(RTF)

Text S2 **Gap recovery procedure.**
(RTF)

Text S3 **MinPathLength procedure.**
(RTF)

Text S4 **Gene family construction procedure.**
(RTF)

Text S5 **Non-metabolic family flagging procedure.**
(RTF)

Text S6 **Bioanalysis of CanOE gene candidates for global orphan enzymes.**
(DOC)
