# Supplementary figures and images for: The CanOE Strategy: Integrating Genomic and Metabolic Contexts across Multiple Prokaryote Genomes to Find Candidate Genes for Orphan Enzymes
Source: PLoS Comput Biol. 2012 May 31;8(5):e1002540. doi: 10.1371/journal.pcbi.1002540 (PMC3364942; doi:10.1371/journal.pcbi.1002540)

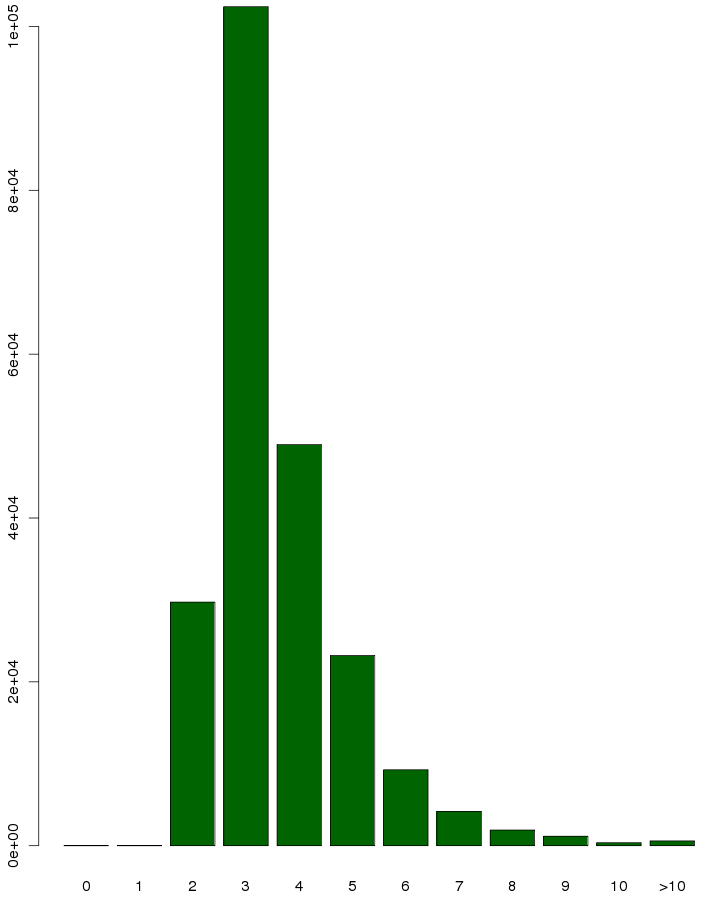

Supplement: Figure S1 — MinPathLength distribution. The number of Known associations for each distinct MinPathLength value (with an imposed maximum of 10) are shown. Note that MPL values of 2 are only possible for Known associations involving multifunctional genes or reactions catalyzed by several gene products (i.e. enzymes with several subunits). (TIFF) [file pcbi.1002540.s001.tiff]

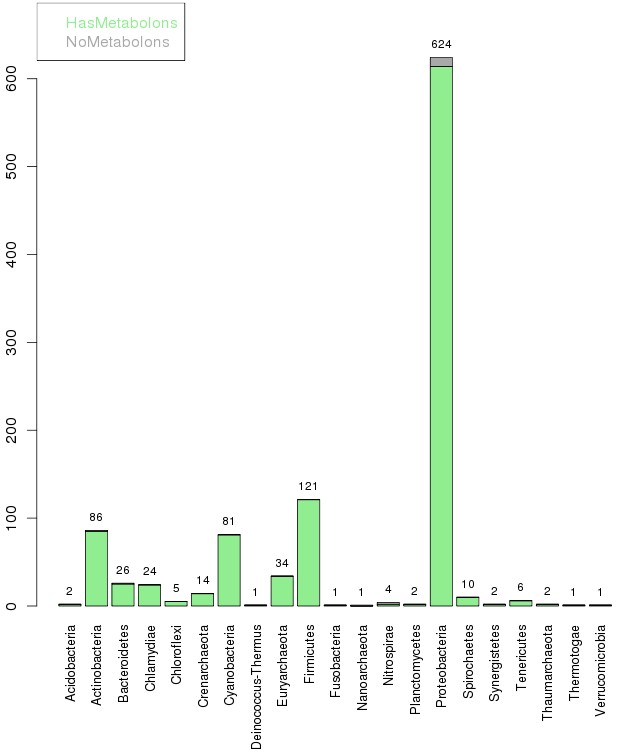

Supplement: Figure S4 — Distribution of prokaryote organisms from the MicroScope database per phylum. The number of MicroScope organisms for each phylum is given. The green fraction of each bar represents the number of organisms that were found to contain metabolons. (TIFF) [file pcbi.1002540.s004.tiff]
