# Supplementary material for: The CanOE Strategy: Integrating Genomic and Metabolic Contexts across Multiple Prokaryote Genomes to Find Candidate Genes for Orphan Enzymes
Source: PLoS Comput Biol. 2012 May 31;8(5):e1002540. doi: 10.1371/journal.pcbi.1002540 (PMC3364942; doi:10.1371/journal.pcbi.1002540)
Supplement: Text S1 — Gene and metabolic graph building. (RTF) [file pcbi.1002540.s009.rtf]

Gene and metabolic graph building
Various small details in the construction of our data graphs are given here :
Gene graph
•	Genomic objects (the basic genomic sequence element defined in MicroScope, see [Vallenet et al., 2006]) corresponding to coding sequences or RNA genes were kept as vertices. Averred artefactual genomic objects are ignored.
•	Neighboring genes (i.e., genes of successive rank along the genome) were interconnected by edges.
•	All fragments of known pseudogenes are interconnected by arcs in order to eliminate their spacing effect
•	Genome-end genes were linked in to respect the circularity of prokaryote genomes, when such information was available.
Using these settings, the number of edges in one of our gene graphs is approximately equal to the number of genes in the studied organism, and the average vertex degree is approximately 2.
Metabolic graph
•	All MetaCyc reactions belonging to at least one MetaCyc pathway are considered as vertices, except those containing “UNKNOWN” compounds.
•	Edges were added between two reactions belonging to a same pathway when:
◦	both reactions share a main compound (as described in the “PATHWAY LAYOUT” field of the MetaCyc pathway)
◦	the reaction directions are compatible with this main compound sharing (i.e. the compound is product of one reaction and substrate of the other)
•	Edges were added between two reactions belonging to different pathways when:
◦	both reactions share a main compound
◦	both pathways are known to be connected, as described in the MetaCyc pathway field “PATHWAY LINKS”
The MetaCyc schema contains 5157 reaction vertices, 5506 reaction-reaction edges, for a mean degree of 2.14.
