# Supplementary material for: The CanOE Strategy: Integrating Genomic and Metabolic Contexts across Multiple Prokaryote Genomes to Find Candidate Genes for Orphan Enzymes
Source: PLoS Comput Biol. 2012 May 31;8(5):e1002540. doi: 10.1371/journal.pcbi.1002540 (PMC3364942; doi:10.1371/journal.pcbi.1002540)
Supplement: Text S2 — Gap recovery procedure. (RTF) [file pcbi.1002540.s010.rtf]

Gap recovery procedure
As said in the Materials & Methods, the Common Connected Components (CCCs) that the CCCPart algorithm returns do not inherently contain gap nodes, as they are CCCs built on graphs with k-partial transitive closures. This means that any two nodes of a same type (gene or reaction) from a CCC may be direct neighbours, or separated by a path of maximal length k in the original graphs (gene graph or reaction graph). Gaps must thus be recovered by walking the original, non-closured graphs between nodes from the CCC. We call “primary” the nodes from a given CCC (genes or reactions). “Primary” edges are those present in the original graphs (gene graph or reaction graph). “Secondary” nodes are recovered gap nodes. “Secondary” edges are edges built from partial transitive closure.
Recovering gene gaps is trivial as they have an almost linear local disposition. We noticed, however, that potentially many alternate paths could correspond to a single primary reaction node pair, especially while experimenting with metabolic networks derived from KEGG maps. To limit the number of possible paths, and thus the number of proposed reaction gaps, we defined several heuristics for gap finding, some based on biological hypotheses :
–	path walking is done exclusively within a given graph type (gene graph or reaction graph)
–	it is unnecessary to search for gaps between primary nodes that are already linked by a primary edge
–	to avoid “loops”, paths are not walked between primary reaction nodes that are linked to the same sets of genes
–	of all possible paths between two primary nodes, the most parsimonious paths (i.e., the shortest) are the most likely; longer paths are discarded
–	amongst the kept shortest paths, paths are scored according to the nature of the reactions (prokaryotic orphan reaction, local orphan reaction in this genome, reaction already present in the genome), favouring paths using reactions already present in the organism (why propose an orphan reaction when an alternate reaction already present in the organism is available ?)
These heuristics can “break” CCCs by effectively removing gap nodes that were “used” in making the CCCs. We thus implemented a post-treatment verification of CCC integrity, removing disconnected nodes and some CCCs. The final gap-filled reaction-pruned product of this process is what we call a genomic metabolon.
In practice, the most obvious results of the pruning described above were: 
–	the elimination of many spurious, non-credible gap paths between primary reactions
–	the elimination of “annotation stars”, i.e. genes associated to multiple, substrate-promiscuous reactions because of lack of annotation precision. Only the reactions actually linked to other reactions in the metabolon were kept.
