# Supplementary material for: The CanOE Strategy: Integrating Genomic and Metabolic Contexts across Multiple Prokaryote Genomes to Find Candidate Genes for Orphan Enzymes
Source: PLoS Comput Biol. 2012 May 31;8(5):e1002540. doi: 10.1371/journal.pcbi.1002540 (PMC3364942; doi:10.1371/journal.pcbi.1002540)
Supplement: Text S3 — MinPathLength procedure. (RTF) [file pcbi.1002540.s011.rtf]

MinPathLength distributions
The MinPathLength (MPL) was designed to be a heuristic measure of “closeness” between a gene and a reaction, be they associated by a known, inferred or potential annotation within a genomic metabolon. Several distance measures are possible between two nodes of the unweighted, undirected graph representing the metabolon. We chose the minimal path length rather than the minimal walk length as the final numerical value would not be different, but more computationally expensive to compute, and less intuitive (why allow backtracking?). Average path or walk lengths would be more complex to compute and would be more relevant to global metabolon connectivity study than to simply determining a distance in the graph.
Unlike during gap finding, edges between genes and reactions can be traversed. However, so as not to generate MPL values of 1, we did not allow edges to be traversed directly between the target gene and reaction, or between the target reaction and other genes associated to it in the same metabolon (to avoid biases generated by multi-subunit enzymes for example). This ensures that the measured MPL captures local connectivity which intuitively represents gene-reaction “closeness”.
To ensure that this heuristic was useful in weighting potential gene-reaction associations, we generated the MPL value distributions for three types of association : Known, Potential, and Imaginary, which corresponds to associations generated between genes and reactions that are neither known nor potential and can be considered as a baseline. These are shown in the figures hereafter.
Known and Imaginary can have an MPL of 2, corresponding to the case when a gene is annotated to at least 2 consecutive reactions. Furthermore, for computational speed reasons, we imposed upper bound of 10  to MPL values.
The different figures show that low MPL values are much more likely that high ones. Known, Potential and Imaginary associations all follow this trend. However, the fraction of Known associations over all associations (Known+Potential+Imaginary) seems to increase with MPL, indicating that the Known distribution has a heavier upper tail. This would suggest that it might not be a good idea to penalise associations with high MPL, as a high-MPL association is more likely to be Known. Manual analysis of several metabolons sporting high-MPL Known associations, however, revealed in each case that the given Known association was spurious, i.e. its inclusion into the metabolon was of limited metabolic relevance.
