# Supplementary material for: The CanOE Strategy: Integrating Genomic and Metabolic Contexts across Multiple Prokaryote Genomes to Find Candidate Genes for Orphan Enzymes
Source: PLoS Comput Biol. 2012 May 31;8(5):e1002540. doi: 10.1371/journal.pcbi.1002540 (PMC3364942; doi:10.1371/journal.pcbi.1002540)
Supplement: Text S4 — Gene family construction procedure. (RTF) [file pcbi.1002540.s012.rtf]

Gene family construction procedure
We wished to use a well-known, proven approach to protein clustering to build protein (and by extension, gene) families. We considered, amongst others, using OrthoMCL [Li 2003] for building our families. OrthoMCL is a tried and tested [Chen ... Roos 2007] program wrapper that calculates protein BLAST e-values normalized for pairs of genomes (in order to reduce phylogenetic biaises) and selects them according to hypothesized orthologue/paralogue relationships. These scores are then passed to the MCL algorithm [van Dongen 2000] which clusters the proteins into (very rarely overlapping) families.
However, at the time, we believed we had found a coding error in the Perl script of the then-available version of OrthoMCL. Moreover, that version of OrthoMCL was incapable of using the BLAST results that were already stored in the MicroScope database. OrthoMCL-DB [Chen 2006] did not have sufficient coverage of the MicroScope prokaryotic genomes (indeed, many of our genomes are private submissions by our collaborators). We thus decided to implement our own version of OrthoMCL, closely mirroring its workings (please note that the latest version of OrthoMCL has completely been reworked by its authors, and also has database support).
BLAST hits were considered for each pair of genomes. A first selection of BLAST hits between proteins was made. Only hits with mincov and maxcov > 70 % were kept (meaning only alignments where at least 70% of the length of each protein matched the other protein were kept). Furthermore, the hits had to be amongst the 5 best hits for either protein. Hits were scored using the following formula :


An average score was established for the pair of genomes, as the average of their gene pair scores.
For construction of the protein similarity matrix to be passed to the MCL algorithm, an additional selection step was included. Hits with e-values higher than 1.10-6, identities lower than 10% or positivities lower than 30%, were ignored. Their score was then normalized by the average score for the genome pair. These normalized scores were passed to MCL.
MCL was run using the following parameters :
-tf “gq(0.65),#knn(175)” -P 12000  -S 1500  -R 2000  -pct 95  -I 1.6
•	gq: minimal edge weight threshold (value chosen after examining edge weight distribution, in order to remove some very rare low-valued edges).
•	knn: for each node, keep the k “nearest neighbors”, i.e. remove node edges so as to keep only the k best (value chosen after manual exploration of the parameter's impact with the MCL tool suite).
•	I: inflation, central parameter for MCL algorithm (value chosen after manual exploration of the parameter's impact with the MCL tool suite)
•	Other parameters are very close to default values.
Resulting families were loaded into the MicroScope database.
