# Supplementary material for: The CanOE Strategy: Integrating Genomic and Metabolic Contexts across Multiple Prokaryote Genomes to Find Candidate Genes for Orphan Enzymes
Source: PLoS Comput Biol. 2012 May 31;8(5):e1002540. doi: 10.1371/journal.pcbi.1002540 (PMC3364942; doi:10.1371/journal.pcbi.1002540)
Supplement: Text S5 — Non-metabolic family flagging procedure. (RTF) [file pcbi.1002540.s013.rtf]

Defining non-metabolic protein families
Many non-metabolic genes exist within prokaryotic genomes, fulfilling activities such molecular transporters, gene expression regulators, DNA duplication and repair, RNA translation, cellular signalling... Within a genomic metabolon, genes responsible for such activities would appear as gap genes; however, it would not make sense to propose them as candidates for reaction gaps if their non-metabolic function is already known. A frequent example of this is the presence of one or more ABC transporters in bacterial operons.
We devised a strategy for flagging gene families as “non-metabolic” to avoid this pitfall. In a first step, we extracted all the InterPro [Apweiler 2000] annotations for all the genes of each of our families. Then, we extracted all the Gene Ontology terms (GOterms) [Ashburner 2000] associated with the InterPro domains. Given the varying levels of annotation present in the families, as well as the non-acyclic nature of the GO term graph, we also extracted all ancestor GO terms, and weighted them by the inverse of the child-ancestor walk distance.
We manually declared some GO terms as “weak-metabolic”, “metabolic”, “weak non-metabolic”, “strong non-metabolic”. 
•	all "molecular_activity" GOterms declared *weak non-metabolic* : 
◦	GO:0005488: binding 
◦	GO:0016247: channel regulator activity 
◦	GO:0042056: chemoattractant activity 
◦	GO:0045499: chemorepellent activity 
◦	GO:0016530: metallochaperone activity 
◦	GO:0060089: molecular transducer activity 
◦	GO:0000988: protein binding transcription factor activity 
◦	GO:0003700: sequence-specific DNA binding transcription factor activity 
◦	GO:0005198: structural molecule activity 
◦	GO:0030528: transcription regulator activity 
◦	GO:0045182: translation regulator activity 

•	all "biological_process" GOterms declared *weak non-metabolic* : 
◦	GO:0065007: biological regulation 
◦	GO:0051234: establishment of localization 
◦	GO:0048519: negative regulation of biological process 
◦	GO:0048518: positive regulation of biological process 

•	all "molecular_activity" GOterms declared *metabolic* : 
◦	GO:0016209: antioxidant activity 
◦	GO:0003824: catalytic activity 
◦	GO:0009055: electron carrier activity 

•	all "molecular_activity" GOterms declared *strong non-metabolic* : 
◦	all GO terms with the word 'transport' in them 
◦	GO:0005215: transporter activity 
◦	GO:0042623: ATPase activity, coupled 
◦	GO:0016820: hydrolase activity, acting on acid anhydrides, catalyzing transmembrane movement of substances 
◦	GO:0004386: helicase activity 
◦	GO:0003774: motor activity 

•	all other "molecular_activity" GOterms : 
◦	GO:0030234: enzyme regulator activity 
◦	GO:0045735: nutrient reservoir activity 
◦	GO:0031386: protein tag 

These declarations were then propagated in the GO graph according to the following rules :
•	a child of a declared metabolic/non-metabolic GO term is respectively metabolic/non-metabolic 
•	precedence: weak non metabolic < metabolic < strong non metabolic (this is amongst others to eliminate transport ATPases & the like) 
•	if a GO term contains 'transport', it is strong non metabolic
•	if a GO term is not declared metabolic or non-metabolic, it is neutral (i.e. we do not have enough information to declare it one or the other)
Each family thus has a list of associated weighted GO terms which can be classified as “metabolic”, “non-metabolic” or  “neutral”. Finally, each family is declared “metabolic”, “non-metabolic” or “neutral”, based on the respective proportions of each type :
•	Metabolic, if the family has metabolic annotations, and over 5% of its metabolic & non-metabolic terms are metabolic ;
•	Non-metabolic, if the family has non-metabolic annotations, and under 5% of  of its metabolic & non-metabolic terms are metabolic ;
•	Neutral otherwise
A total of 616 families (representing 9,402 genes) are declared non-metabolic, 5,935 (184,099 genes) are declared metabolic, and 2,078 (16,320 genes) are neutral. Only the genes from non-metabolic families cannot be proposed as candidate genes; metabolic genes can (obviously) as well as neutral (benefit of doubt). These numbers show that not using non-metabolic families only impacts a small fraction (4.5%) of the available genes, those for which we a almost certain that they are not metabolic. However, this represents an even larger number of false-positive potential associations that are effectively removed.
