# Supplementary material for: The CanOE Strategy: Integrating Genomic and Metabolic Contexts across Multiple Prokaryote Genomes to Find Candidate Genes for Orphan Enzymes
Source: PLoS Comput Biol. 2012 May 31;8(5):e1002540. doi: 10.1371/journal.pcbi.1002540 (PMC3364942; doi:10.1371/journal.pcbi.1002540)
Supplement: Text S6 — Bioanalysis of CanOE gene candidates for global orphan enzymes. (DOC) [file pcbi.1002540.s014.doc]

# Manual bioanalysis of all CanOE predictions for global orphan enzymes

Summary 2

1 - Reaction 1.17.1.1-RXN (bibliographical evidence) 2

2 - Reaction 2-KETO-ADIPATE-DEHYDROG-RXN (implausible prediction) 2

3 - Reaction 2.4.1.121-RXN (implausible prediction) 3

4 - Reaction 4.4.1.25-RXN (no further evidence) 3

5 - Reaction BUTANAL-DEHYDROGENASE-RXN (no further evidence) 3

6 – Reaction CHLOROMALERED-RXN (no further evidence) 3

7 - Reaction CHONDRO-4-SULFATASE-RXN (homology evidence) 3

8 - Reaction DIHYDRONEOPTERIN-MONO-P-DEPHOS-RXN (homology evidence) 4

9 - Reaction FORMYL-COA-HYDROLASE-RXN (implausible prediction) 4

10 - Reaction GALACTONOLACTONASE-RXN (no further evidence) 4

11 – Reaction GLYCINE-AMINOTRANSFERASE-RXN (implausible prediction) 4

12 – Reaction GLYCINE-FORMIMINOTRANSFERASE-RXN (no further evidence) 5

13 – Reaction GUANOSINE-DEAMINASE-RXN (no further evidence) 5

14 – Reaction INDOLE-3-ACETALDEHYDE-OXIDASE-RXN (implausible prediction) 5

15 - Reaction NADNUCLEOSID-RXN (implausible prediction) 5

16 – Reaction OXAMATE-CARBAMOYLTRANSFERASE-RXN (no further evidence) 5

17 - Reaction R265-RXN (homology evidence) 5

18 – Reaction R503-RXN (bibliographical evidence) 6

19 – Reaction R7-RXN (homology evidence) 6

20 – Reaction R83-RXN (bibliographical evidence) 6

21 – Reaction RIBOPHOSPHAT-RXN (homology evidence) 6

22 – Reaction RXN-10032 (implausible prediction) 7

23 – Reaction RXN-10067 (implausible prediction) 7

24 – Reaction RXN-10087 (implausible prediction) 7

25 – Reaction RXN-10446 (implausible prediction) 7

26-27 – Reaction RXN-11518 and RXN-11528 (implausible prediction) 7

28 – Reaction RXN-11566 (implausible prediction) 7

29 – Reaction RXN-11792 (bibliographical evidence) 8

30 – Reaction RXN-12077 (no further evidence) 8

31-32 – Reactions RXN-2005, RXN-2006 (implausible prediction) 8

33 – Reaction RXN-2861 (implausible prediction) 8

34-39 – Reactions RXN-2942, RXN-2944, RXN-2945, RXN-2946, RXN-2947, RXN-2948 (implausible prediction) 8

40 – Reaction RXN-36 (implausible prediction) 8

41 – Reaction RXN-4822 (no further evidence) 8

42 – Reaction RXN-6724 (implausible prediction) 9

43 – Reaction RXN-6883 (implausible prediction) 9

44-45 – Reactions RXN-7746, RXN-7751 (implausible prediction) 9

46 – Reaction RXN-8003 (homology evidence) 9

47 – Reaction RXN-8024 (implausible prediction) 9

48-49 - Reactions RXN-8031, RXN-8032 (implausible prediction) 9

50 – Reaction RXN-8141 (no further evidence) 10

51 - Reaction RXN-8348 (bibliographical evidence) 10

52 – Reaction RXN-8930 (homology evidence) 10

53 – Reaction RXN-8964 (implausible prediction) 10

54-55 – Reactions RXN-9083, RXN-9084 (implausible prediction) 10

56 – Reaction RXN-9222 (implausible prediction) 10

57 – Reaction RXN-9386 (implausible prediction) 11

58-59 – Reactions RXN-9470, RXN-9471 (bibliographical evidence) 11

60 – Reaction RXN-9546 (implausible prediction) 11

61-62 – Reactions RXN-9582, RXN-9586 (implausible prediction) 11

63 - Reaction RXN-9585 (homology evidence) 11

64-65 – Reactions RXN-9867, RXN-9894 (implausible prediction) 11

66 – Reaction RXN0-6368 (implausible prediction) 12

67-71 Reactions RXN1G-98, RXN1G-297, RXN1G-296, RXN1G-295, RXN1G-181 (bibliographical evidence) 12

72 - Reaction RXN3O-1983 (no further evidence) 12

Summary

The list of global orphan enzymes with candidate genes proposed by the CanOE strategy can be accessed at the following URL:

<http://www.genoscope.cns.fr/agc/microscope/metabolism/canoeres.php?Sch_id=MetaCyc&EntrySelection=GlobalOrphanList&GODL=Summary>

Candidate genes and families for the global orphan enzymes found in CanOE metabolons were classified into 4 cases. Their general definitions and the number of global orphan reactions of each type are given in the table below:

| Evidence | Meaning | Number of cases |
| --- | --- | --- |
| Bibliographical evidence | Evidence found in the scientific literature, via sequence similarities or not, backs the CanOE predictions | 12 |
| Homology evidence | High sequence similarity to proteins annotated with similar reactions support the CanOE predictions | 8 |
| No further evidence | No sequence similarity evidence was found to confirm or debunk the CanOE predictions | 11 |
| Implausible prediction | Evidence was found that strongly suggested that the CanOE prediction is improbable | 41 |

“Implausible predictions” are not necessarily incorrect, as the genes involved might have several functions, of which only one is visible in sequence similarity-based or domain-based searches. Furthermore, they can include candidate genes that, even if they visibly do not correspond to the proposed orphan reaction, might still participate in one way or another in the biological process suggested by the metabolons they belong to. In this case, further manual bioanalysis of the said metabolons might reveal other interesting functions to annotate these genes and -why not- alternative candidate genes for the orphan activity in the genes around the metabolon.

Please note that a single metabolon (containing candidate genes AZL_b03570 and AZL_b03590) is responsible for the inclusion in this study of 10 global orphan reactions for which the proposed candidate genes are implausible (as these genes contain permease and regulatory domains, but were not eliminated as non-metabolic since they do not belong to any CanOE gene families).

# **1 - Reaction 1.17.1.1-RXN (bibliographical evidence)**

The family (1718) with the best R->F score gathers proteins sharing high levels of sequence similarity with an experimentally validated protein of ***Yersinia pseudotuberculosis* (Q57174, PubMed: 8071227). The enzymatic activity of this protein corresponds to the MetaCyc reaction 1.17.1.1-RXN. Thus, the prediction of CanOE is correct. This reaction was considered as a global orphan because no proteins are correctly annotated with the corresponding EC number (1.17.1.1) in the MicroScope and UniProt resources.**

# ****2 - Reaction 2-KETO-ADIPATE-DEHYDROG-RXN (implausible prediction)****

**CanOE predicts one family (2998) associated to this global orphan reaction where the metabolons gather 3 reactions for the degradation of 2-aminoadipate, an intermediate metabolite of the lysine degradation pathway. However, the proteins of this candidate family share similarities with RibBA proteins involved in the riboflavin biosynthesis. Thus, the proposed CanOE associations are not plausible, though not impossible, as protein functional promiscuity can hide functions.**

# **3 - **Reaction** 2.4.1.121-RXN (**implausible prediction**)**

CanOE predicts one metabolon with 2 candidate genes for this reaction. The 2 genes share permease or regulatory domains, but as they were not associated to any families, they were not pruned out as non-metabolic genes. In any case, t**he proposed CanOE associations are not plausible.**

# **4 - **Reaction 4.4.1.25-RXN** (no further evidence)**

CanOE predicts 3 families associated to this global orphan reaction (L-cysteate sulfo-lyase, EC:4.4.1.25). Proteins from these gene families share no significant similarities with similar enzymes or domains that are related to the activity. Thus, t**he proposed CanOE associations cannot be confirmed.**

# **5 - **Reaction BUTANAL-DEHYDROGENASE-RXN** (no further evidence)**

CanOE predicts 28 families associated to this orphan reaction (butanal dehydrogenase, EC:1.2.1.57). Proteins from these gene families share similarities with acyl-CoA dehydrogenase domain proteins (EC:1.3.99.-) or 2-dinitropropane dioxygenase (1.13.12.16). In the case of acyl-CoA dehydrogenase, the reaction mechanisms are similar but not completely equivalent to that of EC:1.2.1.57. Thus, t**he proposed CanOE associations cannot be confirmed.**

# **6 – Reaction CHLOROMALERED-RXN (no further evidence)**

One family is predicted by CanOE to be associated to this orphan reaction corresponding to 2-Chloromalehylacetate reduction with NADH as electron donor, without an assigned EC number. This family gathers hypothetical proteins with no significant sequence similarities with known proteins. Thus, t**he proposed CanOE associations cannot be confirmed.**

# **7 - Reaction CHONDRO-4-SULFATASE-RXN (homology evidence)**

This orphan reaction corresponds to the chondro-4-sulphatase reaction (EC:3.1.6.9), where a sulphate ion is removed from dissacharide 4-deoxy-β-D-gluc-4-enuronosyl-(1,3)-*N*-acetyl-D-galactosamine 4-sulfate. CanOE locates 3 metabolons containing this reaction, in which a total of 5 genes are candidates but without any score/rank values since they do not belong to any CanOE families. Among them, the gene Phep_0785 of *Pedobacter heparinus* DSM 2366 shares significant sequence similarity in SwissProt with Arylsulfatases of Eukaryotic origin (EC: 3.1.6.- or 3.1.6.12). Arylsulfatases catalyze reactions similar to chondro-4-sulphatase. Thus, there is **homology** evidence that the CanOE candidate gene can be a correct prediction.

# ****8 - Reaction DIHYDRONEOPTERIN-MONO-P-DEPHOS-RXN (homology evidence)****

**This global orphan reaction corresponds to dihydroneopterin monophosphate dephosphorylase. The family with the highest R->F score corresponds to 7,8-dihydropteroate synthetase (EC:2.5.1.15; folP gene). This family is not a correct candidate. Nevertheless, the second family (3155) gathers proteins harboring a conserved Metal-dependent phosphohydrolase Pfam domain. Thus, there is homology evidence that the CanOE candidate family 3155 might a correct prediction.**

# **9 - Reaction FORMYL-COA-HYDROLASE-RXN (implausible prediction)**

This reaction corresponds to formyl-CoA hydrolase, hydrolyzing formyl-CoA to formate and CoA (EC:3.1.2.10). The candidate family predicted by CanOE corresponds to a NAD-Quinone oxidoreductase associated to EC:1.6.99.5. Thus, t**he proposed CanOE associations are not plausible.**

# **10 - Reaction GALACTONOLACTONASE-RXN (no further evidence)**

This reaction corresponds to 1,4-lactonase (EC:3.1.1.25), that specifically hydrolyzes 1,4-lactones to their corresponding 4-hydroxyacids. The family (2222) with the best R->F score gathers proteins sharing similarities with dihydroxyacid dehydratase (EC:4.2.1.9). The proteins of this family are also candidates for the local orphan activity galactonate dehydratase activity (EC:4.2.1.6) that corresponds to the next reaction step in the galactose/galactonate degradation pathway. Thus, according to homology results, proteins of the family (2222) are likely to catalyze a new type of galactonate dehydratase that do not share any significant similarity with known ones (*i.e.* *dgoD* *E. coli* gene). We can hypothesize that this new type of galactonate dehydratase are bifunctional and also catalyze the orphan lactonase activity.

# **11 – Reaction GLYCINE-AMINOTRANSFERASE-RXN (implausible prediction)**

This reaction corresponds to glycine aminotransferase (EC:2.6.1.4), where an amino group from glutamate is transferred to glyoxylate, producing glycine (from glyoxylate transamination) and oxoglutarate. However, the best candidate family predicted by CanOE gathers 2-dehydro-3-deoxyphosphoheptonate aldolases (EC:2.5.1.54), which catalyze a completely different reaction.

# ****12 – Reaction GLYCINE-FORMIMINOTRANSFERASE-RXN (no further evidence)****

CanOE predicts a metabolon in *Cloacamonas acidaminovorans* with 2 candidate genes for the reaction **GLYCINE-FORMIMINOTRANSFERASE-RXN**. These genes encode hypothetical proteins with no significant similarities with known proteins. Thus, t**he proposed CanOE association cannot be confirmed.**

# **13 – Reaction GUANOSINE-DEAMINASE-RXN (no further evidence)**

CanOE predicts the gene LC705_00798 of *Lactobacillus rhamnosus* Lc705 as a candidate for this reaction. This protein shares no significant similarities with known enzymes or domains. Thus, t**he proposed CanOE candidate cannot be confirmed.**

# **14 – Reaction INDOLE-3-ACETALDEHYDE-OXIDASE-RXN (implausible **prediction**)**

CanOE predicts one metabolon with 2 candidate genes for this reaction. The 2 genes share permease or regulatory domains. Thus, t**he proposed CanOE associations are not plausible.**

# **15 - Reaction NADNUCLEOSID-RXN (implausible prediction)**

**This reaction corresponds to NAD+ nucleotidase associated to EC:3.2.2.5. The candidate family predicted by CanOE encodes a hypothetical protein with low similarities (*i.e.* 28 % amino acid identity) with Poly-beta hydroxybutyrate polymerases (EC:2.3.1.-) which catalyze a completely different reaction. Thus, the proposed CanOE candidate is not plausible.**

# **16 – Reaction OXAMATE-CARBAMOYLTRANSFERASE-RXN (no further evidence)**

See manuscript “Case study” section.

# **17 - Reaction R265-RXN (homology evidence)**

This reaction corresponds to the reduction of cyclohex-1,5-diene-1-carbonyl-CoA with an undefined electron donor. The candidate family proposed by CanOE harbors one gene (RPA0656/*badC* of *Rhodopseudomonas palustris* CGA009) that shares similarities with NAD+ dependent alcohol dehydrogenases which correspond to a similar redox reaction. **Thus, there is homology evidence that the CanOE candidate gene is a correct prediction.**

# **18 – Reaction R503-RXN** **(bibliographical evidence)**

This reaction corresponds to diketodeoxyinositol hydrolase, which catalyzes the hydrolysis of D-2,3-diketo-4-deoxy-*epi*-inositol to 5-dehydro-2-deoxy-D-gluconate in the context of the myo-inositol degradation pathway. The family (2089) with the best R->F score gathers proteins sharing high level of similarities with the IolD protein of ***Bacillus subtilis* that was experimentally validated as catalyzing this hydrolase activity (PubMed: 18310071). Thus, the prediction of CanOE is correct. This reaction was considered as a global orphan because the BioCyc pathologic software used in MicroCyc was not able to correctly project this reaction in *Bacillus subtilis* by the lack of a precise EC number and compatible product descriptions.**

# **19 – Reaction R7-RXN (homology evidence)**

This reaction corresponds to the deacetylation of 3-hydroxy-5-oxohexanoyl-CoA, yielding (S)-3-hydroxybutanoyl-CoA and Acetyl-CoA (acetyl group transfer to CoA molecule). The reaction is associated to the partial EC 2.3.1-. Two proteins of **the family (619, genes BB4703 and RALSYv4_40684) with the highest R->F score are predicted as** candidates. They show significant similarities with acetyltransferase subunits of pyruvate dehydrogenase (EC:2.3.1.12) and succinyltransferase subunits of oxoglutarate dehydrogenase (EC:2.3.1.61). These reactions are similar to the orphan reaction R7-RXN (acetyl group transfer). **Thus, there is homology evidence that the CanOE candidate genes may be correct predictions.**

# **20 – Reaction R83-RXN (bibliographical evidence)**

This reaction corresponds to the dephosphorylation of 2-hydroxy-3-keto-5-methylthio-1-phosphopentene in the context of *S*-methyl-5-thio-α-D-ribose 1-phosphate degradation pathway. The family (2320) with the best R->F score gathers proteins sharing high level of similarities with an experimentally validated Enolase phosphatase E-1 of *Klebsiella oxytoca* (PubMed: 8227040). This protein is a bifunctional enzyme that catalyzes the enolization of 2,3-diketo-5-methylthiopentyl-1-phosphate into the intermediate 2-hydroxy-3-keto-5-methylthiopentenyl-1-phosphate and the subsequent dephosphorylation to form the acireductone 1,2-dihydroxy-3-keto-5-methylthiopentene. This second dephosphorylation reaction corresponds to the reaction R83-RXN. **Thus, the prediction of CanOE maybe correct.**

# **21 – Reaction RIBOPHOSPHAT-RXN (homology evidence)**

This reaction corresponds to the dephosphorylation of 5-amino-6-(5-phospho-D-ribitylamino)uracil to 5-amino-6-(D-ribitylamino)uracil in the context of flavin biosynthesis pathway. CanOE predicts several candidate families for this reaction. Among them, the family 2244 gathers ThiL proteins that catalyze the phosphorylation of thiamine phosphate in the context of the thiamine biosynthesis pathway (*i.e.* a reaction similar to RIBOPHOSPHAT-RXN). In *Buchnera aphidicola* APS, the *thiL* gene is present in an operon with other genes involved in flavin biosynthesis. However, in *B.* *aphidicola* APS, the thiamine biosynthesis pathway is no longer functional due to an extreme genome reduction process. Given this and the fact that the *thiL* gene is maintained intact in the context of flavin biosynthesis, we can hypothesize that ThiL may be a good candidate for the RIBOPHOSPHAT-RXN. **Thus, there is evidence that the CanOE candidate family 2244 could be correct prediction.**

# **22 – Reaction RXN-10032 (implausible prediction)**

CanOE predicts one metabolon with one candidate gene for this reaction. This gene has InterPro hits with domains for signal transduction. Thus, t**he proposed CanOE association is not plausible.**

# **23 – Reaction RXN-10067 (implausible prediction)**

This reaction corresponds to phosphomevalonate decarboxylase (decarboxylation of mevalonate 5P to isopentenyl phosphate; no EC assigned). The best candidate family proposed by CanOE shares high levels of sequence similarity with Ribonuclease J proteins. Thus, t**he proposed CanOE association is not plausible.**

# **24 – Reaction RXN-10087 (implausible prediction)**

This reaction corresponds to chloromuconolactone dehalogenase. The candidate family predicted by CanOE shares domains for periplasmic ligand binding. Thus, t**he proposed CanOE association is not plausible.**

# **25 – Reaction RXN-10446 (implausible prediction)**

This reaction corresponds to hydroxylation of salycilate to produce gentisate. The candidate CanOE family gathers proteins of the fumarylacetoacetate hydrolase family. Thus, t**he proposed CanOE association is not plausible.**

# **26-27 – Reaction RXN-11518 and RXN-11528 (implausible prediction)**

These reactions correspond to 7-methylxanthine demethylase, where 7-methylxanthine is demethylated yielding xanthine and formaldehyde. The best candidate family proposed by CanOE shares weak similarities with XdhC proteins that assist in molybdopterin insertion into xanthine dehydrogenase. Thus, t**he proposed CanOE association is not plausible.**

# **28 – Reaction RXN-11566 (implausible prediction)**

This reaction corresponds to the reductive condensation of L-aspartate semialdehyde and putrescine yielding carboxyspermidine, in the context of variant II of the Spermidine biosynthesis pathway (MetaCyc: PWY-6559). The candidate genes proposed by CanOE share similarities with enzymes that are not related to the RXN-11566 reaction. Thus, t**he proposed CanOE association is not plausible.**

# **29 – Reaction RXN-11792 (bibliographical evidence)**

This reaction corresponds to the α-D-glucopyranosyl-diphosphoundecaprenol glucosyltransferase activity in the context of xanthan/acetan biosynthesis. CanOE predicts one metabolon in *Xylella fastidiosa* 9a5c containing this reaction. A gene neighbor (XF2360) of this metabolon is a homolog of the *gumM* gene of *Xanthomonas campestris* that was experimentally demonstrated to be essential for the activity RXN-11792 (PubMed: 9537354). **Thus, the CanOE metabolon prediction is credible.**

# **30 – Reaction RXN-12077 (no further evidence)**

This reaction corresponds to oxalate oxidoreductase reaction, with oxalate that is oxidized to 2 molecules of CO2 with generic ferredoxin as redox pair. The proposed CanOE family gathers hypothetical proteins with no significant similarities to known proteins. Thus, t**he proposed CanOE associations cannot be confirmed.**

# **31-32 – Reactions RXN-2005, RXN-2006 (implausible prediction)**

These reactions correspond to 3-oxo-3-phenylpropionyl-CoA thiolase and benzoyl-CoA thioestherase respectively, and are involved in the benzoate biosynthesis pathway. The predicted candidate gene by CanOE corresponds to a transcriptional regulator of benzoate catabolism. Thus, t**he proposed CanOE association is not credible.**

# **33 – Reaction RXN-2861 (implausible prediction)**

This reaction corresponds to methanol dehydrogenase (oxidation of methanol to formaldehyde with electron transfer to cytochrome molecules). The proposed candidate families share similarities with proteins that are not related to the RXN-2861 reaction.

# **34-39 – Reactions RXN-2942, RXN-2944, RXN-2945, RXN-2946, RXN-2947, RXN-2948 (implausible prediction)**

CanOE predicts one metabolon with 2 candidate genes for these reactions. The 2 genes share permease or regulatory domains. Thus, t**he proposed CanOE associations are not plausible.**

# **40 – Reaction RXN-36 (implausible prediction)**

This reaction corresponds to 4-acetamidobutyrate deacetylase involved in putrescine degradation. The best candidate family proposed by CanOE shares similarities with tartrate dehydrogenases. Thus, t**he proposed CanOE associations are not plausible.**

# **41 – Reaction RXN-4822 (no further evidence)**

CanOE predicts one metabolon with 1 candidate gene for this reaction. The proposed gene encodes a hypothetical protein with no significant similarities to known proteins. Thus, t**he proposed CanOE association cannot be confirmed.**

# **42 – Reaction RXN-6724 (implausible prediction)**

This reactions corresponds to benzoyltransferase (EC:2.3.1.196). The predicted candidate gene corresponds to a transcriptional regulator of benzoate catabolism. Thus, t**he proposed CanOE association is not credible.**

# **43 – Reaction RXN-6883 (implausible prediction)**

This reaction corresponds to uniquinone oxidase involved in aerobic respiration. The proposed candidate families share similarities with proteins not related to the RXN-6883 reaction. Thus, t**he proposed CanOE associations are not plausible.**

# **44-45 – Reactions RXN-7746, RXN-7751 (implausible prediction)**

CanOE predicts one metabolon with 3 candidate genes for these reactions. One of these genes encodes a transcriptional regulator. The two others may correspond to artefactual CDSs. Thus, t**he proposed CanOE associations are probably not correct.**

# **46 – Reaction RXN-8003 (homology evidence)**

This reaction corresponds to cis-4-hydroxy-D-proline oxidase (oxidation of 2-cis-4-hydroxy-D-proline to 1-pyrroline-4-hydroxy-2-carboxylate; EC:1.5.1.-). CanOE predicts one metabolon in *Vibrio sp.* EX25 with 3 candidate genes for this reaction. The gene VEA_003672 shares similarities with D-amino-acid oxidoreductases. **Thus, there is homology evidence that the CanOE candidate gene is a correct prediction.**

# **47 – Reaction RXN-8024 (implausible prediction)**

This reaction corresponds to phytoene dehydrogenase, where all-trans phytofluene is oxidized to all-trans carotene in the context of carotene biosynthesis. Candidate families proposed by CanOE corresponds to lycopene cyclases (further steps in carotene biosynthesis). Thus, t**he proposed CanOE associations are not credible.**

# **48-49 - Reactions RXN-8031, RXN-8032 (implausible prediction)**

These reactions correspond to the hydratation of 6-carboxyhex-2-enoyl-CoA producing 3-hydroxypimelyl-CoA and to the acetyl group transfer from 3-oxopimeloyl-CoA to CoA, yielding glutaryl-CoA and AcCoA. Both reactions are involved in the benzoyl-CoA degradation pathway. The single candidate gene proposed by CanOE encodes a ketoacyl-CoA reductase (mechanistically different). Thus, t**he proposed CanOE associations are not plausible.**

# **50 – Reaction RXN-8141 (no further evidence)**

CanOE predicts one metabolon with 1 candidate gene for this reaction. The proposed CanOE gene encodes a hypothetical protein with no significant similarities with known proteins. Thus, t**he proposed CanOE association cannot be confirmed.**

# **51 - Reaction RXN-8348 (bibliographical evidence)**

This reaction corresponds to molybdenum:molybdopterin ligase in the context of molybdenum cofactor biosynthesis. A metabolon in *Kytococcus sedentarius* DSM 20547 contains 4 gap genes. Among them, one gene (Ksed_19980) is similar to *moeA* of *E. coli* K-12 that was experimentally demonstrated to mediate molybdenum ligation to molybdopterin (*i.e.* RXN-8348 activity, PubMed: 15632135). **Thus, the CanOE metabolon prediction is reliable.**

# **52 – Reaction RXN-8930 (homology evidence)**

This reaction corresponds to a biuret amidohydrolase (EC:3.5.1.84). CanOE predicts a metabolon in *Agrobacterium vitis* S4 where one gap gene (Avi_5651) shares sequence similarity with peroxyureidoacrylate/ureidoacrylate amidohydrolase RutB proteins. **Thus, there is homology evidence that the CanOE candidate gene is a correct prediction.**

# **53 – Reaction RXN-8964 (implausible prediction)**

This reaction corresponds to succinyl-CoA:L-malate CoA transferase. The proposed candidate families share similarities with proteins not related to the RXN-8964 reaction.

# **54-55 – Reactions RXN-9083, RXN-9084 (implausible prediction)**

CanOE predicts one metabolon with 2 candidate genes for these reactions. The 2 genes share permease or regulatory domains. Thus, t**he proposed CanOE associations are not credible.**

# **56 – Reaction RXN-9222 (implausible prediction)**

This reaction corresponds to the condensation of 4-hydroxybenzoate with heptaprenyl diphosphate to produce 3-heptaprenyl-4-hydroxybenzoate in the context of ubiquinol-7 biosynthesis. The proposed candidate families share similarities with proteins not related to the RXN-9222 reaction.

# **57 – Reaction RXN-9386 (implausible prediction)**

The proposed candidate families share similarities with proteins not related to the RXN-9386 reaction (Glutamyl-tRNA^Glx^ synthetase reaction, no EC number assigned).

# **58-59 – Reactions RXN-9470, RXN-9471 (bibliographical evidence)**

These two reactions take place in the streptomycin biosynthesis pathway. A metabolon in *Streptomyces griseus* NBRC 13350 gathers 2 gap genes (*strF* and *strG*) that are candidates for these two reactions. These genes were previously hypothesized to be involved in the streptomycin biosynthesis (PubMed: 1654502). **Thus, the CanOE metabolon prediction may be correct.**

# **60 – Reaction RXN-9546 (implausible prediction)**

This reaction corresponds to the enoyl-CoA reductase involved in stearate biosynthesis. The CanOE candidate family shares similarities with glutamine synthetases. **Thus, the CanOE metabolon prediction is not plausible.**

# **61-62 – Reactions RXN-9582, RXN-9586 (implausible prediction)**

These reactions are associated to the hypothetical isomerization of kanosamine 6-phosphate to 3-amino-3-deoxy-D-fructose-6-phosphate and to 5-deoxy-5-amino-3-dehydroquinate dehydratation in the context of 3-amino-5-hydroxybenzoate biosynthesis from kanosamine.

The proposed candidate families share similarities with proteins that are not related to these reactions.

# **63 - Reaction RXN-9585 (homology evidence)**

This reaction is associated to the dephosphorylation of 3,4-dideoxy-4-amino-D-*arabino*-heptulosonate 7-phosphate to 5-deoxy-5-amino-3-dehydroquinate in the context of 3-amino-5-hydroxybenzoate biosynthesis from kanosamine. CanOE detects a metabolon in *Amycolatopsis mediterranei* U32 harboring a candidate gene (AMED_0629) similar to known phosphoglycolate phosphatases. **Thus, there is homology evidence that the CanOE candidate gene is a correct prediction.**

# **64-65 – Reactions RXN-9867, RXN-9894 (implausible prediction)**

These reactions correspond to a chloromuconate cycloisomerase activity and a chloromuconate cycloisomerase activity. The candidate gene proposed by CanOE encodes a putative periplasmic ligand binding protein. **Thus, the CanOE predictions are not plausible.**

# **66 – Reaction RXN0-6368 (implausible prediction)**

The metabolons predicted by CanOE gather 3 reactions for the tetrahydromonapterin biosynthesis with one orphan reaction (Reaction RXN0-6368). The candidate CanOE family shares high levels of similarity with FolE proteins that hydrolyze GTP to give the 7,8-dihydroneopterin triphosphate, which is the starting point for the tetrahydromonapterin biosynthesis. Thus, the associations proposed by CanOE are inaccurate.

# **67-71 Reactions RXN1G-98, RXN1G-297, RXN1G-296, RXN1G-295, RXN1G-181 (bibliographical evidence)**

These 5 orphan reactions are simultaneously found in the same metabolons by CanOE. They give a chemical description of the Claisen-type condensation (condensase) which takes place in the final step of the mycolic acid synthesis in Mycobacteriaceae. For these 5 reactions, the candidate CanOE family (2450) with the best R->F score gathers proteins from Corynebacterineae which harbor domains that are commonly found in polyketide synthases. Furthermore, these proteins share high levels of sequence similarity with two proteins (Uniprot entries: Q6T306, Q8NLR7) which were proposed as candidates for the condensase activity based on experimental evidence (PubMed: 14695899). Thus, the prediction of CanOE is consistent with previously-established experiment-based hypotheses.

# **72 - Reaction RXN3O-1983 (no further evidence)**

CanOE predicts a metabolon in *Thermus thermophilus* HB8 with 2 candidate genes for the reaction RXN3O-1983. These genes encode hypothetical proteins with no significant similarities with known proteins. Thus, t**he proposed CanOE association cannot be confirmed.**
